# A retrospective study of dynamic navigation system-assisted implant placement

**DOI:** 10.1186/s12903-023-03481-2

**Published:** 2023-10-14

**Authors:** Lijuan Ma, Mingjun Ye, Mingle Wu, Xiaolei Chen, Shan Shen

**Affiliations:** 1 Department of Stomatology, Affiliated Stomatological Hospital of Jinan University (Daliang Hospital Shunde District Foshan City), Foshan City, 528399 Guangdong Province China; 2 School of Stomatology, Jinan University, Guangzhou City, 510620 Guangdong Province China; 3 Department of Stomatology, The First Affiliated Hospital of Jinan University, No. 613, Huangpu Avenue West, Tianhe District, Guangzhou, 510632 Guangdong Province China

**Keywords:** Digital navigation technology, Dynamic navigation system, Implant surgery, Real-time navigation

## Abstract

**Background:**

To evaluate the accuracy of implant placement assisted by a dynamic navigation system, as well as its influencing factors and learning curve.

**Methods:**

At Macao We Care Dental Center, 55 cases of implant placement using dynamic navigation were retrospectively evaluated. To evaluate their accuracy, the apex, tip, and angle deviations of preoperatively planned and postoperatively placed implants were measured. The effects of the upper and lower jaws, different sites or lateral locations of dental implants, and the length and diameter of the implants on accuracy were analyzed, as well as the variation in accuracy with the increase in the number of surgical procedures performed by dentists.

**Results:**

The implant had an apex deviation of 1.60 ± 0.94 mm, a tip deviation of 1.83 ± 1.03 mm, and an angle deviation of 3.80 ± 2.09 mm. Statistical differences were observed in the tip deviation of implants at different positions based on three factors: jaw position, lateral location, and tooth position (P < 0.05). The tip deviation of the anterior teeth area was significantly greater than those of the premolar and molar areas. There were no statistically significant differences in apex deviation, tip deviation, or angle deviation between the implants of different diameters and lengths (P > 0.05). There were significant differences in the angle deviation between the final 27 implants and the first 28 implants. Learning curve analysis revealed that angle deviation was negatively correlated with the number of surgical procedures, whereas the regression of apex deviation and tip deviation did not differ statistically.

**Conclusions:**

The accuracy of dynamic navigation-assisted dental implants meets the clinical needs and is higher than that of traditional implants. Different jaw positions, lateral locations, and implant diameters and lengths had no effect on the accuracy of the dental implants guided by the dynamic navigation system. The anterior teeth area had a larger tip deviation than the posterior teeth area did. As the number of dynamic implantation procedures performed by the same implant doctor increased, the angle deviation gradually decreased.

## Background

Implant dentures are commonly used to restore missing teeth, and the success and long-term survival rates of implants are important for implant doctors. According to Monje A et al. [1], implants placed too buccally can lead to alveolar bone loss. This can affect the restoration of the upper crown. Although doctors can customize the abutment to fix this issue, it increases the patient’s chair time and additional costs. [2] In addition, there are important anatomical structures in the upper and lower jaws, such as the maxillary sinus and the inferior alveolar nerve. [3] Research indicates that 6.89% of complications are related to poor three-dimensional implant placement, which can cause damage to adjacent anatomical structures. [4] For example, implants may accidentally displace into the maxillary sinus, submandibular space, or nasal cavity. [5–7] The concept of implantation guided by restoration emphasizes the importance of accurate three-dimensional implant positioning.

Computer-assisted implantation technology, including static guidance and dynamic navigation technology, was introduced in 1995. Numerous studies have shown that both static guidance and dynamic navigation provide better accuracy than freehand implantation. [8–11] In 2018, during the ITI consensus discussion, it was reported that static computer-assisted implant surgery had a global platform deviation of 1.2 mm, a global apical deviation of 1.5 mm, and an angle deviation of 3.5° on average, meeting safety standards in most cases. [12] However, static guides have certain limitations: fabricating preoperative guide plates takes time and incurs cost; intraoperative surgical plans cannot be altered in real-time; there is a risk of bone burn; patient’s mouth opening affects the procedure; specific implant surgical tools are required. [13, 14] Dynamic navigation technology can overcome these limitations associated with static guides. [8, 15]

The accuracy of early-developed dynamic navigation systems was mostly limited to in vitro model studies, and some systems like RoboDent are no longer used in clinical practice. [16] Recently, several meta-analyses have evaluated the in vivo accuracy of dynamic navigation systems. Wei et al. [17] evaluated five dynamic navigation systems (X-Guide, Navident, AqNavi, ImPlaNav, and IRIS-100) and found a global platform deviation of 1.02 mm, global apical deviation of 1.33 mm, and angular deviation of 3.59°. JORBA-GARCíA et al. [18] evaluated nine dynamic navigation systems and reported an average angular deviation of 3.68° and a global platform deviation of 1.03 mm based on five clinical studies in the literature. SCHNUTENHAUS et al. [19] evaluated four commercial dynamic navigation systems and found a global platform deviation of 1.00 mm, global apical deviation of 1.33 mm, and angular deviation of 3.7°. Overall, the reported accuracies in these studies are similar. However, some scholars have observed deviations exceeding 1 mm and suggest following a safe distance of 2 mm for implantation. It should be noted that most recent literature on the accuracy of commercial dynamic navigation systems is based on in vitro models. While in vitro studies allow for better control over variables and elimination of interference factors found within the human body, there are significant differences between oral cavity tissues in vitro and in vivo, including their precision and mobility.

Therefore, the accuracy reported in literature needs to be interpreted cautiously, and further clinical research with increased sample sizes is required. [20] Currently available clinical research on commonly used dynamic navigation systems is limited to a small number of surgical teams. It is necessary to evaluate whether other surgical teams can achieve similar clinical outcomes to analyze the accuracy and feasibility of these systems. In this study, we retrospectively analyzed the implantation accuracy of dynamic navigation-assisted implants. We made an invalid assumption that dynamic navigation implant surgery cannot achieve satisfactory clinical accuracy. Additionally, we discussed the factors influencing the accuracy of dynamic navigation systems and explored the initial learning curve of these systems.

## Methods

### Research participants

The Medical Ethics Committee of Jinan University approved this study (grant number: JNUKY-2022-044) and all patients who participated in the study signed an informed consent form for dental implant surgery. From January 2020 to October 2021, 55 cases of dynamic navigation implantation were retrospectively analyzed. Patients ranged in age from 29 to 75 years, with an average age of 53.47 ± 12.83 years. This study included 18 men and 37 women. With a total of 55 implants, there were 7 cases of anterior teeth, 15 cases of premolars, and 33 cases of molars. The inclusion criteria were as follows: (1) the patient was at least 20 years old, (2) CBCT images showed that the bone in the missing tooth area was healing well, and (3) the patient provided informed consent and was in good general health. Exclusion criteria:1) history of smoking; 2) history of bruxism or moderate/severe periodontitis; 3) history of diabetes, history of head-and-neck radiotherapy or chemotherapy 5 years ago, and other systemic diseases or lifestyle habits that affect implant synostosis; (4) moderate or severe mouth opening restrictions; (5) based on the patient’s medical records, bone augmentation and intraoperative flap were performed during surgery; and (6) missing preoperative or postoperative CBCT data.

### Surgical procedure

**Fig. 1:**
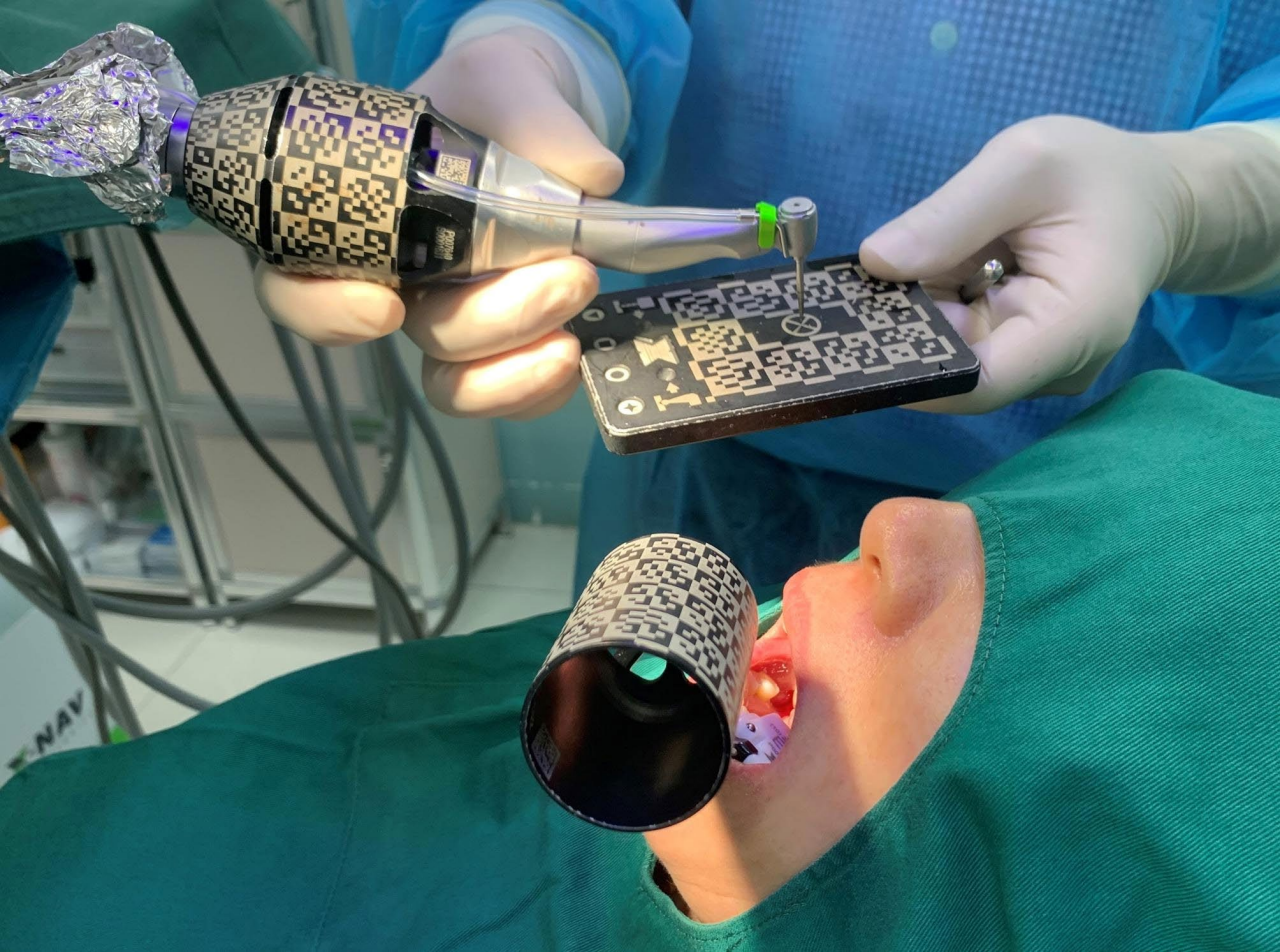
A preoperative calibration check was conducted by placing the selected drill on the through-hole plate and measuring its length

Prior to the CBCT scan, the hot bath-treated X-Clip was correctly positioned in the patient’s mouth, which is typically on the opposite side of the same jaw in the implant area. A CBCT scan was performed on the patient, with the X-Clip placed accurately and steadily. The CBCT parameters were set as follows: (1) field of view (FOV) included all registration devices and surgical sites; (2) FOV was 6 cm in diameter and 6 cm in height; (3) voxel size of the CBCT image was 0.4 mm. Digital imaging and communications in medicine (DICOM) data obtained from the CBCT scan were loaded into the dynamic navigation system, and DTX Studio software was used to design the implant’s three-dimensional position. The X-clip was positioned concurrently and tagged within the image. The chair position and light were adjusted after the patient was seated on a dental chair. The toothless area was anesthetized via local infiltration with 0.8 ml of 4% articaine. Skin was prepared and draped routinely. In addition, standardization and calibration were performed prior to navigation. The registration steps were as follows: (1) the surgical instruments were calibrated to ensure that the pattern ends were within the scope of the navigation camera, (2) the X-Clip was attached to the patient tracker, and (3) a preoperative calibration check was conducted by placing the selected drill on the through-hole plate and measuring its length (Fig. 1). Then, for calibration inspection, the drill was used to touch the three reference balls on the surface of the X-Clip; (4) the X-Clip was reattached to the patient’s teeth; (5) the navigation camera was positioned so that the pattern end of the surgical instruments and tracker were within its range; (6) a system check was conducted to ensure that the X-Clip was correctly positioned, that the head tracker was properly connected to the head, and that the camera was in the correct position. In this step, the doctor can determine whether the position of the drill tip is correct by comparing the head movement to the virtual drill on the navigation screen. If the input was confirmed as correct, the navigation view was displayed on the monitor. Based on the real-time navigation screen, Nobel Active implants were placed in the intended location, followed by the placement of a closure screw or healing cap. The patient underwent a second CBCT after surgery.

### Accuracy analysis

**Fig. 2:**
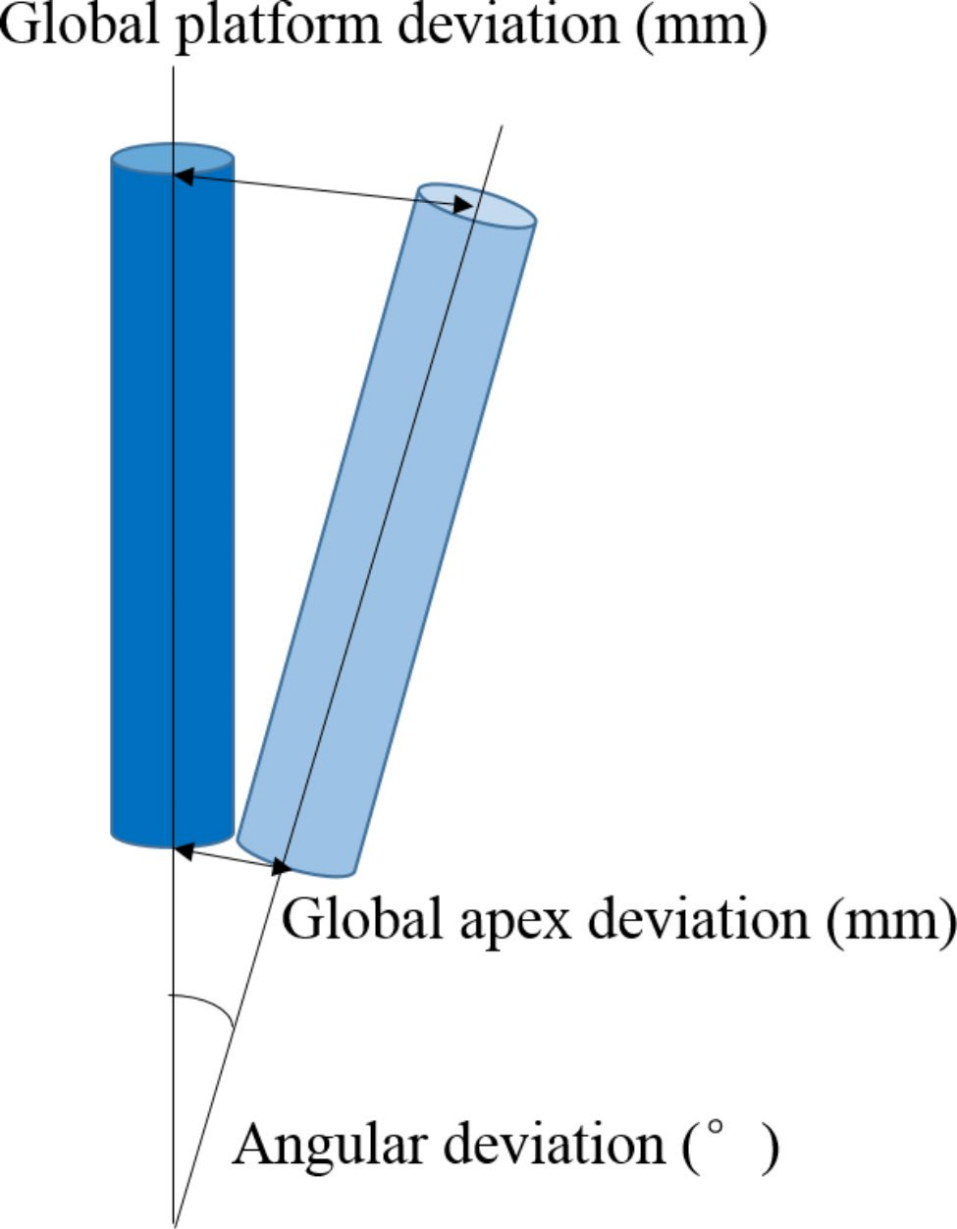
Model diagram of accuracy analysis

Actual and planned implant deviations were analyzed on the same computer by the same analyst using X-Guide internal analysis software. The apex, tip, and angle deviations were the primary deviation indicators. All data were measured and recorded three times on average. Apex deviation: linear displacement (mm) between the actual implant and the planned implant at the center of the implant platform. Tip deviation: linear displacement (mm) at the end between the actual and planned implants. Angle deviation: The angle offset (°) between the actual implant and the planned implant’s central axis (Fig. 2).

The data was imported into the SPSS 27.0 statistical analysis software. Kolmogorov-Smirnov test was used to determine the normality of the data distribution. Normal distribution of measurement data are expressed as mean ± standard deviation (x ± s).

### Effect of implantation sites on accuracy

The angle, apex, and tip deviations of different jaw positions (maxillary and lower jaw), lateral locations (left and right), and implantation sites (anterior teeth, premolars, and molars) were compared using a multi-factor analysis of variance. Differences were considered statistically significant at P < 0.05. Multiple comparisons of the indicators with statistically significant differences were performed.

### Effect of implant characteristics on accuracy

The implant deviations of different diameters and lengths were analyzed using one-way ANOVA, and the difference was deemed statistically significant when P < 0.05. For pairwise tests involving indicators with statistically significant differences, the LSD method was used.

### Learning curve analysis

The 55 dental implants were divided into two surgical stages: Group A (28 dental implants) and Group B (27 dental implants), based on the order of the operation. The 55 patients were arranged sequentially according to the time of consultation. Using the sequence number as the abscissa and the angle, apex, and tip deviation as the ordinate, the Graphpad Prism 8.0.2 software was used to plot the regression curve. Using linear regression, the change in implant deviation as the number of surgical procedures increased, was observed. The inspection level was set as α = 0.05. P < 0.05 was considered statistically significant.

## Results

### Analysis of the accuracy of dental implant with dynamic navigation system

The data for apex deviation (Z = 1.064, P = 0.207), tip deviation (Z = 0.693, P = 0.723), and angle deviation (Z = 0.724, P = 0.671) were determined to be approximately normally distributed using the Kolmogorov-Smirnov test.

The primary indicators of implant deviation (Average deviation and maximum and minimum values of deviation) are apex deviation (1.60 ± 0.94) mm (0.38–3.94 mm), tip deviation (1.83 ± 1.03) mm (0.32–4.79 mm), and angle deviation (3.80 ± 2.09)° (0.81–8.64°). The greater the accuracy of dental implants using dynamic navigation, the lower the measured deviation value.

### The effects of implantation sites on the accuracy of dental implant with dynamic navigation system

**Fig. 3:**
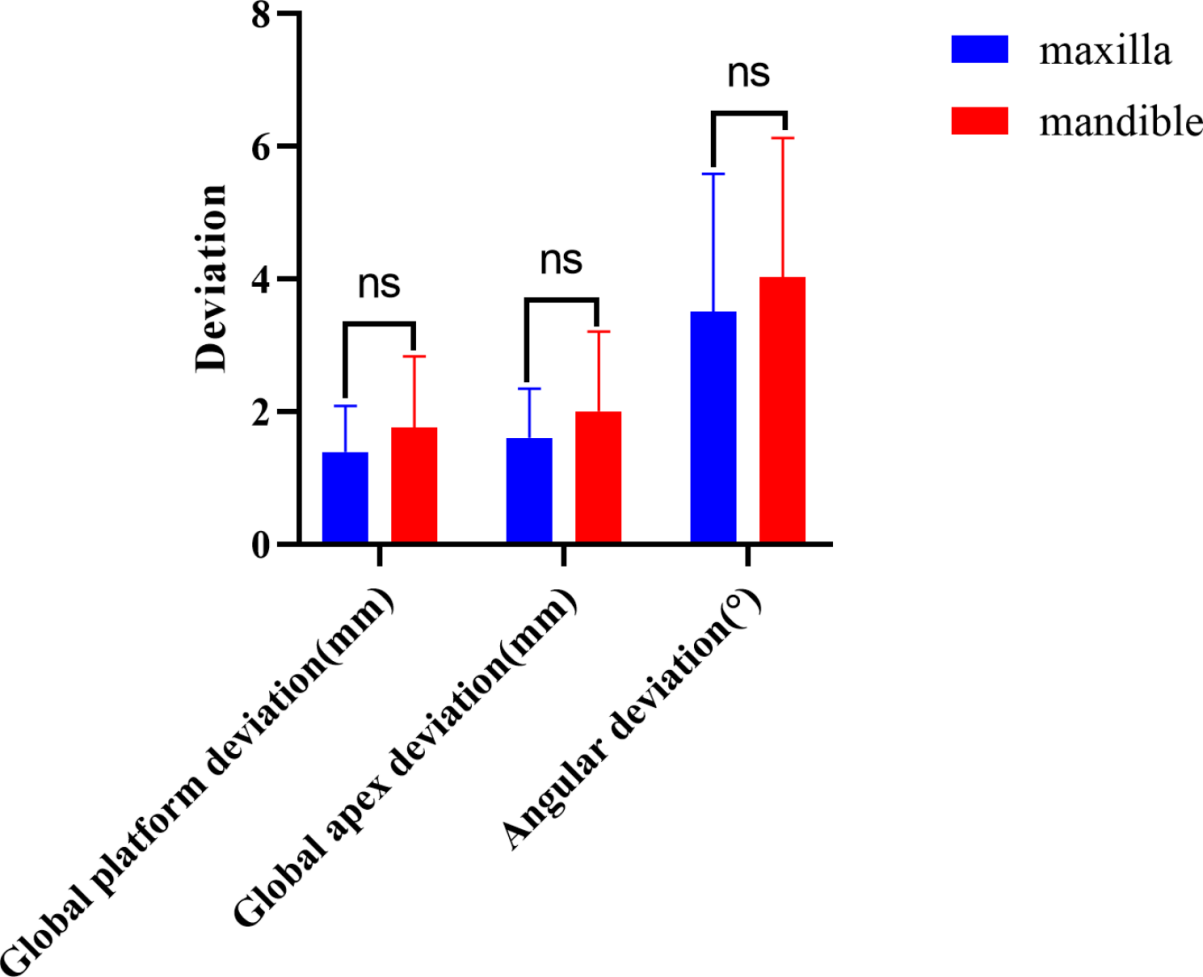
Comparison of the placement accuracy of maxillary and mandibular implants

**Fig. 4:**
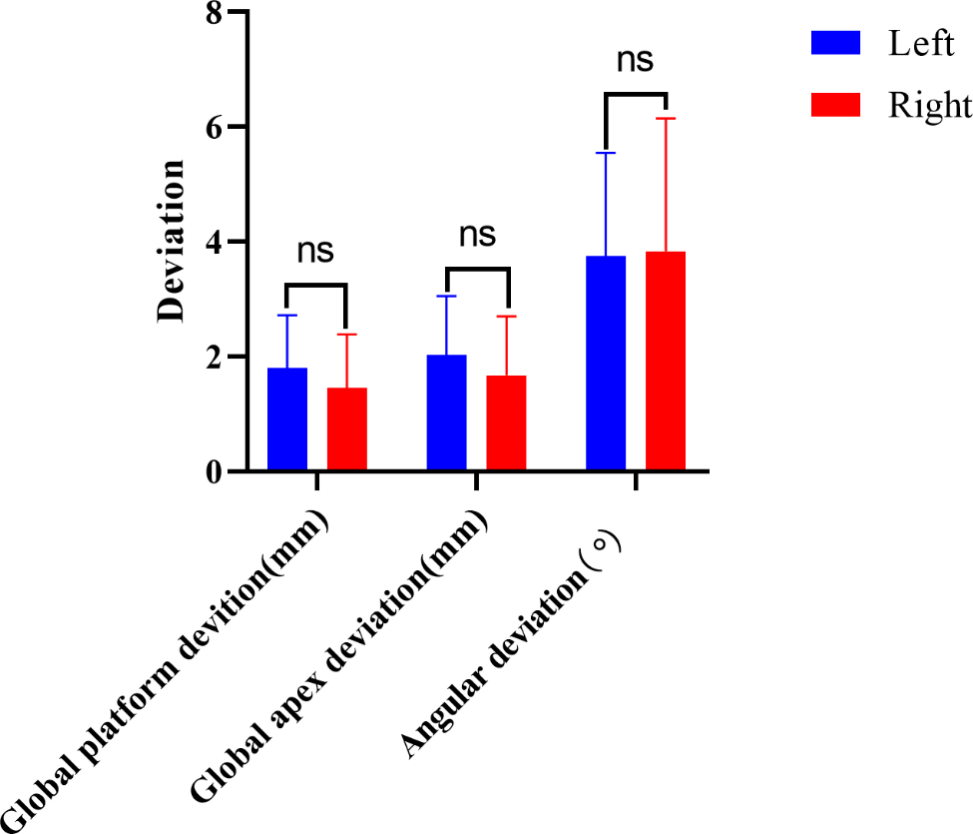
Comparison of implant placement accuracy of different lateral implants

The variance analysis of the three factors revealed that among the three factors—different jaw positions, lateral locations, and tooth positions–only the tip deviation of dental implants in different tooth positions exhibited a statistically significant difference (P < 0.05), while the others did not. There was no interaction between these factors (Table 1; Figs. 3 and 4).

**Table 1:** Comparison of tip deviations at different tooth positions

Tooth position (I)	Tooth position (J)	Difference value of the mean (I-J)	*P*
Anterior teeth area	Premolar area	1.097	0.007
	Molar area	0.773	0.033
Premolar area	Anterior teeth area	-1.097	0.007
	Molar area	-0.324	0.225
Molar area	Anterior teeth area	-0.773	0.033
	Premolar area	0.324	0.225

Post hoc multiple comparisons revealed statistically significant differences between the tip deviation of the anterior teeth area and the premolar area as well as between the anterior teeth area and the molar area (P < 0.05). Deviation of the tip of the anterior teeth was significantly greater than that of the premolar and molar teeth. There was no statistically significant difference between the premolar and posterior tooth areas (P > 0.05) (Table 1).

### The effect of implant characteristics on the accuracy of dental implant with dynamic navigation system

There were no statistically significant differences between implant diameters and lengths for apex deviation, tip deviation, or angle deviation (P > 0.05) (Tables 2 and 3).

**Table 2:** Influence of different implant diameters on main implant deviations (x ± s)

Mean deviations	Diameter of the implant (mm)			F	*P*
	3.5 and below	4.3	5.0		
Apex deviation (mm)	1.83 ± 1.07	1.42 ± 0.78	1.78 ± 1.13	1.165	0.320
Tip deviation (mm)	1.98 ± 1.26	1.74 ± 0.94	1.88 ± 0.99	0.276	0.760
Angle deviation (°)	4.29 ± 2.36	3.78 ± 2.10	3.12 ± 1.57	0.939	0.398

**Table 3:** Effect of implant of different lengths on the major deviations of implants (x ± s)

Mean deviations	Length of the implant (mm)				F	*P*
	8.5	10	11.5	13 and above		
Apex deviation (mm)	1.28 ± 0.61	1.83 ± 1.05	1.50 ± 1.02	1.72 ± 0.93	0.712	0.549
Tip deviation (mm)	1.38 ± 0.73	1.94 ± 1.26	1.79 ± 0.98	2.02 ± 1.07	0.796	0.502
Angle deviation (°)	3.35 ± 1.70	4.26 ± 2.08	3.42 ± 2.04	4.14 ± 2.38	0.649	0.587

### Statistical description of the learning curve

In this study, there was a statistically significant difference between the angle deviation of the last 27 implants and the first 28 implants (t = 2.206, P = 0.032), but there was no statistically significant difference between apex deviation and tip deviation (P > 0.05) (Table 4).

**Table 4:** Comparison of the accuracy of the first 28 implants to the last 27 implants (x ± s)

Sequence of clinical visit	Apex deviation (mm)	Tip deviation (mm)	Angle deviation (°)
Group A	1.75 ± 0.83	1.98 ± 0.92	4.73 ± 2.65
Group B	1.54 ± 1.03	1.84 ± 1.25	3.28 ± 2.20
t	0.815	0.476	2.206
*P*	0.418	0.636	0.032

**Fig. 5:**
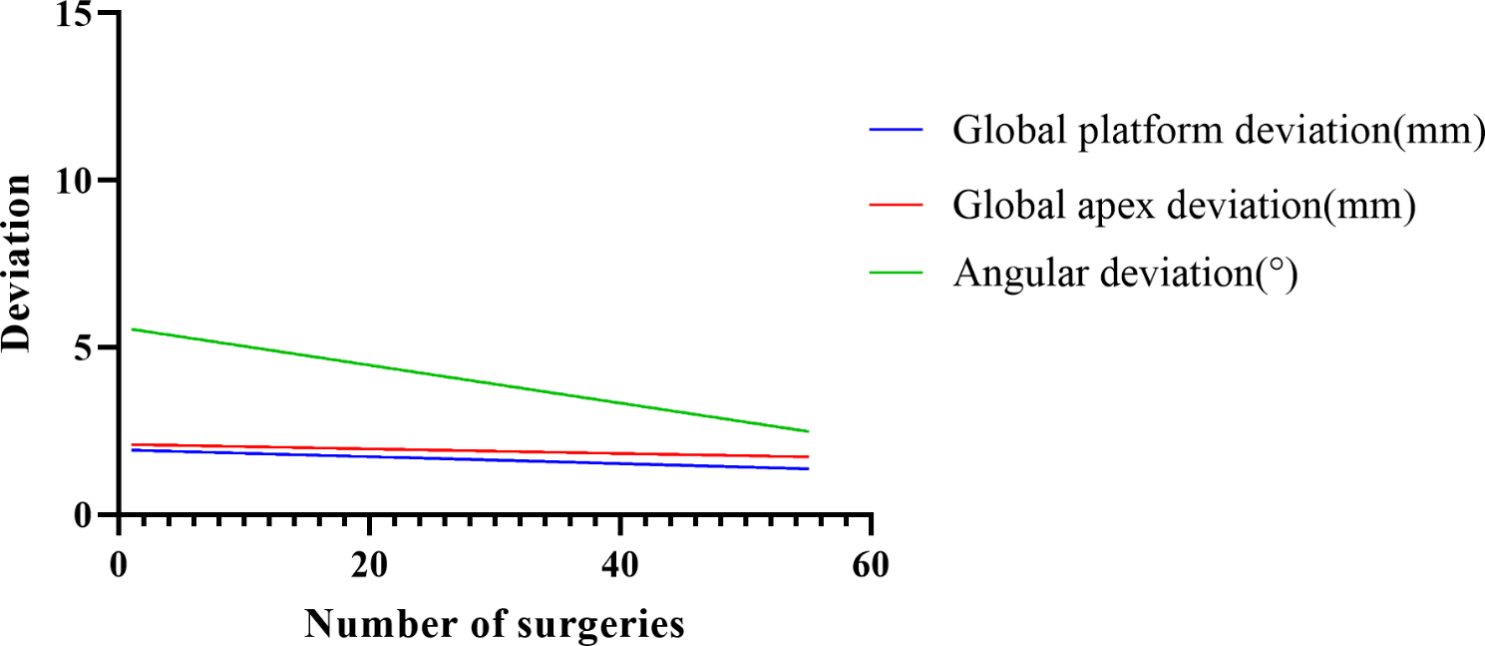
Regression curve and vertex, end and angle deviation of dynamic navigation guided planting in different time periods

The angle deviation (F = 7.842, P = 0.007) was negatively correlated with the number of surgical procedures. The regression equation was angle deviation=-0.05667*number of surgical procedures + 5.604, R^2^ = 0.129. There was no statistical difference between the apex deviation (P = 0.191) and the tip deviation (P = 0.462) regressions (Fig. 5).

## Discussion

With the rise of commercial dynamic navigation systems, there has been growing interest among dentists in utilizing these technologies. However, there is still a lack of clinical studies that thoroughly investigate the accuracy of dynamic navigation systems. It is therefore important to delve into topics such as accuracy, factors that influence accuracy, and the learning curve associated with dynamic navigation implants. By doing so, dentists can gain a comprehensive understanding of dynamic navigation systems and their potential benefits.

### Analysis of dynamic navigation assisted-surgery accuracy

The results of this study showed that the dynamic navigation system had an apex deviation of 1.60 ± 0.9 mm, tip deviation of 1.83 ± 1.03 mm, and angle deviation of 3.80 ± 2.09°. It is important to note that these measurements were obtained from in vitro model studies.[21] The average bias (tip deviation/angle deviation) in these studies was 0.38 mm/0.89°, which was relatively low in comparison to the average deviations observed in in vivo studies, which were 1.83 mm/3.80°. [21] This difference can be attributed to factors such as mouth opening and closing, mucosal mobility, and visual field restrictions in the posterior teeth region. [22, 23]

In a prospective cohort study conducted by Block et al., they found that the precision of the dynamic navigation system was higher than what was observed in our study. [2] The angle deviation reported by Block et al. was 2.97 ± 2.09°, apex deviation was 1.16 ± 0.59 mm, and tip deviation was 1.29 ± 0.65 mm.[2] It is worth mentioning that this disparity in accuracy could be attributed to various factors including study design, surgical approach, type of analysis software utilized, and the inclusion of a larger number of dental implant cases. Overall, while our study demonstrated relatively high dental implant accuracy with the dynamic navigation system under investigation, it is essential to consider the limitations associated with comparing in vitro model studies to in vivo studies and take into account various factors that may affect accuracy outcomes in clinical settings.

Due to the limited sample size in this study, it is not possible to draw definitive conclusions. However, when comparing the accuracy of the dynamic navigation system with traditional implantation methods performed without assistance, it does seem that the dynamic navigation method offers superior accuracy.

### Analysis of the influencing factors of dynamic navigation system accuracy

In this study, no statistically significant differences were found in implant accuracy between different lateral locations or between the upper and lower jaws, which aligns with previous findings. [8, 13] However, there was a significant difference in tip deviation between the anterior and posterior teeth areas. This difference may be attributed to limitations imposed by the optical tracking system and the visual field, as well as variations in bone density between these regions. The number of dynamic navigation implants performed by dentists can also impact accuracy, as a dull drill needle may lead to increased deviation when drilling into dense bone. It is important to note that using traditional drilling techniques may also affect implant survival rate. [24, 25] De Oliveira et al. [26] found significant variations in bone density among different anatomical regions of the oral cavity, with the anterior mandible having the highest average bone density, followed by the anterior maxilla, posterior mandible, and posterior maxilla. Due to the hardness of bone in the anterior teeth area, the drilling needle tends to turn towards areas of lower resistance, resulting in larger tip deviations in implant placement. Additionally, when dentists place the drilling needle on the alveolar crest, its movement can be affected by sliding and deflection caused by compact bone. [27, 28] Moreover, as more dynamic navigation implants are performed over time, there may be a decrease in drill needle sharpness leading to increased deviation when drilling into dense bone. This highlights some of the challenges and factors that can influence accuracy during dynamic navigation implantation.

During the placement of dental implants in the anterior teeth area, surgeons typically utilize two monitoring windows: the navigation monitoring screen on the computer and direct vision with the naked eye. This simultaneous engagement in two visual tasks can lead to a competition for visual attention resources, ultimately resulting in decreased precision during the operation under computer navigation monitoring.

### Effect of implant characteristics on the dynamic navigation system accuracy

There were no statistically significant differences observed in terms of implant length, apex deviation, tip deviation, or angle deviation between different implants. However, it was found that implants with a length of 8.5 mm displayed higher accuracy compared to implants of other lengths. This could be attributed to the precise preparation of planting holes with specific depths and the surgeon’s ability to have strong real-time adjustable control over the three-dimensional orientation of the implant. On the other hand, when the length of the implant exceeded 13 mm, there was an increase in average deviation at the end of the implant reaching 2.02 ± 1.07 mm. Existing literature has indicated that longer implants tend to exhibit a greater error deviation from the preoperative plan. [29] However, it is important for implant doctors to consider maintaining a safety margin of at least 2 mm between the end of the implant and important anatomical structures. [12].

### Learning curve analysis of dynamic navigation system

Surgical skills tend to improve with clinical experience, which can be visualized as a learning curve. [30] It has been observed that the learning curve for dynamic navigation systems reaches a plateau after approximately five procedures. [24] In the later stages of the learning curve, advancements become slower until a plateau is eventually reached. Block reported that achieving proficiency in dynamic navigation technology required approximately 20 cases. [31] In this study, statistically significant differences in angle deviation were found between the final 27 implants and the initial 28 implants. Linear regression analysis was conducted to assess the learning curve, revealing differences in the regression of angle deviation but no statistical difference in the regressions of apex deviation and tip deviation. This indicates that the dentist in this study was able to control apex and tip deviations to some extent. There are several factors that may contribute to this control: (1) The dentist had almost 10 years of experience with dental implants and possessed extensive theoretical knowledge and practical experience. During surgery, the dentist developed improved control over planting timing and fulcrum utilization. (2) The dentist participated in a two-month in vitro model training period, which provided a fundamental understanding of the dynamic navigation system. Moreover, the regression curve showed that as the number of implant procedures increased, angle deviation decreased, suggesting that dentists’ control over angle deviation improved over time with an increased number of implant procedures. According to our study, surgeon experience emerged as a significant factor influencing the accuracy of dynamic navigation systems. [8] While previous studies have indicated that accuracy is not significantly correlated with physician experience based on in vitro model studies, [16, 32, 33] it is important not to generalize these findings to in vivo studies. Zhan et al. [34] discovered that utilizing dynamic navigation systems significantly improved implant placement accuracy for students with no previous implantation experience during training. They also observed an improvement in accuracy during the last five procedures compared to previous procedures. Wu et al. [35] found no statistically significant difference in implantation accuracy between experienced and less experienced surgeons, nor between two experienced surgeons in the dynamic navigation group. However, it should be noted that all surgeons in their study received training in dynamic navigation systems prior to surgery. This suggests that the surgeons reached a certain level of proficiency and were at the end of their respective learning curves, indirectly highlighting the significance of the learning curve.

## Conclusion

The accuracy of dynamic navigation-assisted implantation meets the clinical requirements and surpasses the precision of conventional implantation techniques. This technology offers advantages such as improved clinical operation, real-time feedback on implantation site conditions, and a relatively simple digital workflow. It has the potential to be widely promoted and adopted to enhance the overall quality of oral implant procedures. In order to enhance accuracy, it is crucial for doctors to gain proficiency with the system through repeated studies and training prior to clinical practice. It is important to note that accuracy may vary between implantation sites due to factors such as bone density and visual field limitations. The limitations of this study, including its small sample size and focus on single missing tooth cases with gap implantation, necessitate further clinical research to confirm the application of dental implant therapy.

## Data Availability

The data that support the findings of this study are available from the corresponding author, upon reasonable request.
